# The epidemiology of malaria and anaemia in the Bonikro mining area, central Côte d’Ivoire

**DOI:** 10.1186/1475-2875-13-194

**Published:** 2014-05-27

**Authors:** Astrid M Knoblauch, Mirko S Winkler, Colleen Archer, Mark J Divall, Milka Owuor, Raoul M Yapo, Pokou A Yao, Jürg Utzinger

**Affiliations:** 1 Department of Epidemiology and Public Health, Swiss Tropical and Public Health Institute, P.O. Box CH-4002 Basel, Switzerland; 2 University of Basel, P.O. Box, CH-4003 Basel, Switzerland; 3 SHAPE Consulting Ltd, Pretoria 0062, South Africa; 4 University of Kwa Zulu Natal, Durban 4041, South Africa; 5 Newcrest Mining Limited, Immeuble Danny Centre, II Plateaux, Vallon, Abidjan, Côte d’Ivoire

**Keywords:** Malaria, Anaemia, *Plasmodium falciparum*, Mining, Private sector, Côte d’Ivoire

## Abstract

**Background:**

The epidemiology of malaria and anaemia is characterized by small-scale spatial and temporal heterogeneity, which might be influenced by human activities, such as mining and related disturbance of the environment. Private sector involvement holds promise to foster public health, including the prevention and control of malaria and anaemia. Here, results from a cross-sectional epidemiological survey, conducted in communities that might potentially be affected by the Bonikro Gold Mine (BGM) in Côte d’Ivoire, are reported.

**Methods:**

In December 2012, a cross-sectional survey was carried out in seven communities situated within a 20-km radius of the BGM in central Côte d’Ivoire. Capillary blood samples were obtained from children aged six to 59 months. Samples were subjected to a rapid diagnostic test (RDT) for *Plasmodium falciparum* detection, whilst haemoglobin (Hb) was measured to determine anaemia. Additionally, mothers were interviewed with a malaria-related knowledge, attitudes and practices questionnaire.

**Results:**

A total of 339 children and 235 mothers participated in the surveys. A positive RDT for *P. falciparum* was found in 69% of the children, whilst 72% of the children were anaemic (Hb <11 g/dl). *Plasmodium falciparum* infection was significantly associated with anaemia (odds ratio (OR) 7.43, 95% confidence interval (CI) 3.97-13.89), access to a health facility (OR 5.59, 95% CI 1.81-17.32) and age (OR 0.04, 95% CI 0.01-0.12; youngest (six to 11 months) *versus* oldest (48-59 months) age group). Less than a quarter of mothers knew that malaria is uniquely transmitted by mosquitoes (22.3%, 95% CI 16.8-27.7%). Misconceptions were common; most of the mothers believe that working in the sun can cause malaria.

**Conclusions:**

Malaria and anaemia are highly endemic in the surveyed communities around the BGM project area in Côte d’Ivoire. The data presented here provide a rationale for designing setting-specific interventions and can be utilized as a benchmark for longitudinal monitoring of potential project-related impacts due to changes in the social-ecological and health systems.

## Background

The extractive industry, especially mining, is an important economic sector in many African countries [1]. Meanwhile, Africa is the continent most heavily affected by malaria, which drains the social and economic development [2-4]. Knowledge about whether and to what extent the epidemiology of malaria is altered in the face of extractive industry projects in high endemicity areas is limited. Mining and other natural resources development and management projects are believed to influence local disease patterns, including malaria, through a variety of proximal and distal factors. For example, environmental manipulation to accommodate project-related infrastructure developments can create favourable habitats for malaria vectors, most importantly *Anopheles gambiae*[5,6]. Mining projects attract workers and camp followers who might live in poorly constructed houses with inadequate sanitation. Lack of drainage, along with peri-urban water bodies (e.g. swamps), might increase vector breeding and human-vector contact [7,8]. On the other hand, mining projects create opportunities for the prevention and control of malaria as the companies have an interest in keeping their workforce healthy, and thus productive. This may, at the same time, positively impact on surrounding communities, as has been shown in the Zambian copperbelt in the 1930s and 1940s, and elsewhere [9,10]. Moreover, corporate social responsibility has become part of international good practice for companies operating in resource-constraint settings [11]. In remote areas in developing countries, where health systems are notoriously weak and understaffed, the success of national control efforts depends on the coordination and communication among different stakeholders (e.g. government, non-governmental organizations, international institutions and the private sector) [12,13]. In such settings, the private sector can play a pivotal role in fostering public health, as emphasized by the World Health Organization (WHO) [14].

The Bonikro Gold Mine (BGM) project in central Côte d’Ivoire is planning malaria control efforts to mitigate health risks of the workforce, but is simultaneously exploring opportunities to support sustainable development initiatives in the communities surrounding the BGM project. However, there was a paucity of data regarding malaria prevalence, vector and parasite species composition and insecticide resistance in the project area. As this limited the ability to design control interventions, studies were commissioned on the following grounds: (i) the malaria prevalence is showing small-scale spatial heterogeneity [15-18]; (ii) a deeper understanding of the epidemiology of malaria in the study area will facilitate the development of targeted interventions [19]; and (iii) a detailed characterization of the baseline situation allows for longitudinal monitoring and evaluation of project impacts [20], and malaria-specific interventions. Additionally, the concurrent assessment of anaemia was considered important, as it serves as an indicator for general health and well-being [21-23].

Against this background, the following activities were undertaken: (i) a malaria and anaemia prevalence survey among children aged six to 59 months to determine the prevalence of *Plasmodium falciparum* and the magnitude of anaemia; (ii) a knowledge, attitudes and practices (KAP) survey related to malaria among mothers; and (iii) an entomology and insecticide susceptibility survey in order to identify effective insecticides for potential indoor residual spraying (IRS) and other vector control interventions. Here, the findings of the malaria and anaemia prevalence surveys and mothers’ KAP study are presented. Detailed findings of the entomology and insecticide resistance surveys will be communicated elsewhere.

## Methods

### Ethical considerations

Ethical clearance was obtained from the ‘Comité National d’Ethique et de la Recherche’ of Côte d’Ivoire (reference no. 01/MSLS/CNER-dkn). Communities were given detailed information about the purpose and procedures of the study, the extent of their involvement, the right to withdraw anytime without further obligation and to receive free treatment based on the results of the measurements or tests done. Written informed consent was obtained from all participating mothers in the questionnaire survey before conducting any interviews, which included the approval to take capillary blood samples from their children aged six to 59 months in a mobile clinic. Individuals with a *P. falciparum* infection or anaemia were treated according to national guidelines, free of charge. Children who had a positive RDT result for *P. falciparum* were given artesunate/amodiaquine (Camoquin Plus; Pfizer, New York, USA) for uncomplicated malaria (if no prior treatment was received). Anaemic children were provided with iron supplements (Ferrostran; Teofarma, Valle Salimbene, Italy) and severe cases were referred to health care centres. Independent of the test results, all participating children aged above three years received a multi-vitamin suspension (Alvityl; Laboratoires URGO, Chenôve Cedex, France).

### Study area

**Figure 1:**
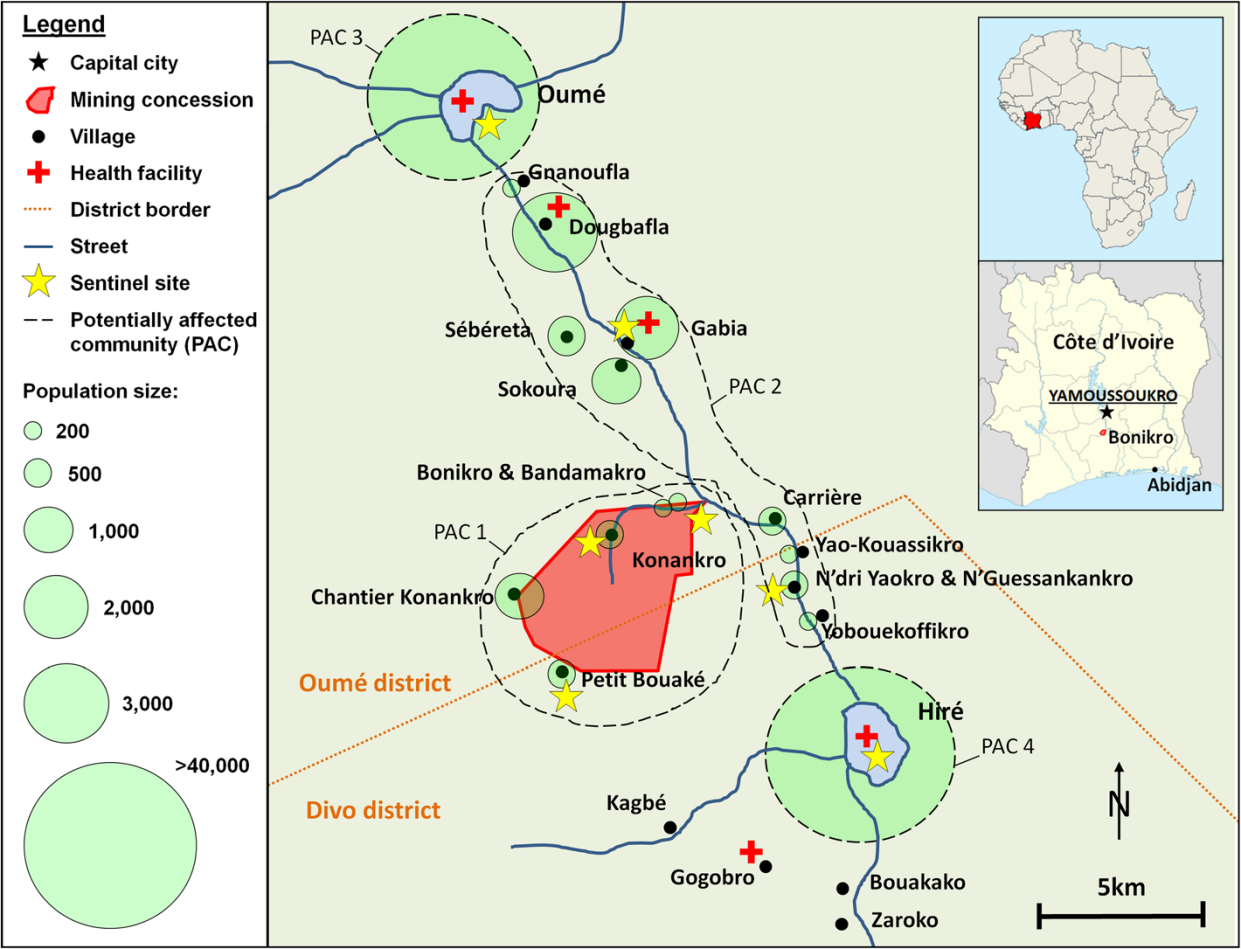
Study area and surveyed sentinel sites, including estimated population sizes, Côte d’Ivoire, 2012.

The study was carried out in the health districts of Oumé and Divo in central Côte d’Ivoire. The study area comprised two major towns, Oumé and Hiré, and several smaller villages around the BGM project (between latitudes 6°11’ and 6°22’ N and longitudes 5°17’ and 5°24’ W). A map of the study area, including estimated population sizes for 2011, is provided in Figure 1. The total population residing in the surveyed sentinel sites is estimated at 12,000, whereas in Oumé and Hiré, the respective neighbourhood population, not the entire urban population, was counted.

### Study design and household selection

A cross-sectional household survey was carried out in December 2012, at the end of the rainy season. Based on *P. falciparum* prevalence rates found among young children in previous studies conducted in central and south-central Côte d’Ivoire [16,17,24,25] and in consultation with local health staff, the prevalence of malaria in the study area was estimated at 70% (p = 0.7). With a 95% level of confidence and a precision of 5%, the sample size was calculated at 336.

The sampling methodology applied was adapted to the specific project setting and was based on a sentinel survey approach. Given the considerable demographic differences in the study area (see population sizes in Figure 1), sampling proportional to size was not considered appropriate, as smaller villages in proximity to the project area were believed to be more directly affected by project activities than larger towns further away. Instead, villages and towns were grouped into potentially affected communities (PACs), purposely defined as specific geographical entities that are considered to be similarly impacted by project-related activities (see Figure 1, dashed line) [20]. For the BGM project, the following PACs were defined: (1) the immediate project area (potentially affected through infrastructure developments and resettlement); (2) the access road between Oumé, Hiré and the project site (potentially affected by migration and increased traffic); and (3) and (4) the urban centres of Oumé and Hiré, respectively (potentially affected through migration and potential economic shifts and representative of most of the BGM workforce). Within these four PACs that take into consideration specific project exposure, seven sentinel sites were randomly selected; thee in PAC 1, two in PAC 2, and one each in PAC 3 and PAC 4.

Within sentinel sites, a random sampling procedure at the unit of the household was applied. Firstly, trained interviewers were sent in four random directions from a central location [20]. Secondly, interviewers counted the number of houses up to the border of the sentinel site. Thirdly, the field manager determined, through a random procedure, the first household to be interviewed in each direction. The interviewer then proceeded to interview the next household until the targeted number (minimum of 25 households per day) was achieved. The two criteria for household inclusion were: (i) household inhabited by at least one child under the age of five years; and (ii) willingness of the child’s mother to respond to a questionnaire.

### Survey activities

Mothers were first interviewed using a pretested KAP questionnaire. The questionnaire focussed on household demographics, malaria awareness and knowledge, modes of transmission, preventive measures, including ownership and utilization of insecticide-treated nets (ITNs), IRS and treatment-seeking behaviour for malaria. All questions were closed-ended and drawn from international standard surveys, such as Demographic and Health Surveys (DHS), to facilitate subsequent comparison with regional and national data.

Once the KAP questionnaire was completed, participating mothers were invited to bring their children aged below five years to a mobile clinic, located at a central place in each sentinel site. Capillary blood samples were collected from children aged six to 59 months. Following this, tests were conducted to determine haemoglobin (Hb) levels using a portable HemoCue device (HemoCue® Hb 201 System; HemoCue AB, Ängelholm, Sweden) and presence of *P. falciparum* infection using an RDT (Paracheck Pf device cassette; Orchid Biomedical Systems, Goa, India).

### Statistical analysis

Data were recorded using Microsoft Excel version 2010 (Microsoft Corporation; Redmond, USA) and EpiData version 3.1 (EpiData Association; Odense, Denmark). Statistical analyses were done using Stata version 10 (StataCorp.; College Station, USA). Prevalence rates were averaged for background characteristics, such as age and sex. Adjusted odds ratios (ORs) with their corresponding 95% confidence intervals (CIs) were calculated using multivariate logistic regression analysis for child and household attributes associated with *P. falciparum* infection and anaemia. P-values below 0.05 were considered statistically significant.

## Results

### Study compliance and respondents’ characteristics

**Figure 2:**
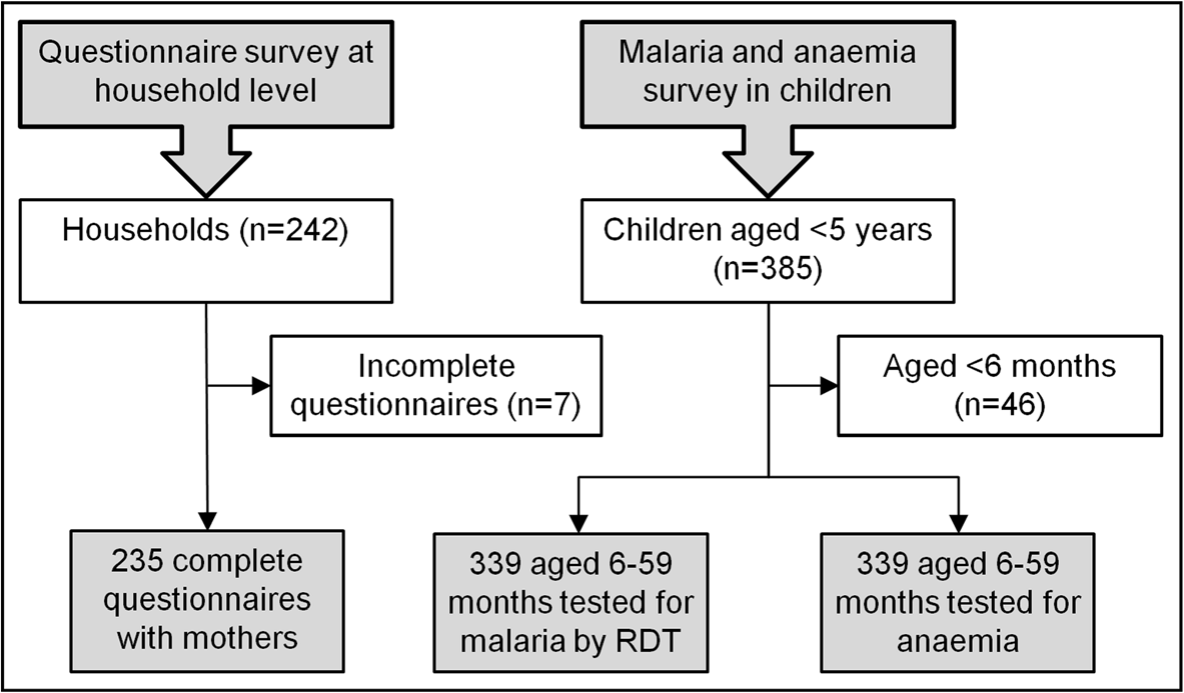
Study compliance.

Overall, 574 individuals participated in the study (Figure 2). There were 242 households visited and 235 mothers had complete data records from the questionnaire survey. Without exception, mothers visited the mobile clinics, readily accompanied by their children. Overall, there were 385 children aged below five years (on average 1.44 children per mother). Among these, 339 children had a blood sample subjected to an RDT for *P. falciparum* and measuring Hb level. The mean age of the mothers interviewed was 27.9 years (standard deviation (SD), 7.4 years). Slightly more than half of the mothers (123/235, 52.3%) had no formal education, whereas 80 mothers (34.0%) had primary education and the remaining 32 mothers (13.6%) attained secondary education or higher.

### *Plasmodium falciparum* infection

The prevalence of *P. falciparum* in children aged six to 59 months was 69.0% (95% CI 64.1-74.0%) (Table 1) and varied by age. The youngest age group (six to 11 months) showed the lowest infection prevalence (47.8%; 95% CI 32.8-62.8%), whilst the oldest age group had the highest prevalence (48-59 months; 83.9%, 95% CI 74.5-93.3%). Similar prevalences were found for boys and girls.

**Table 1:** Malaria prevalence by rapid diagnostic tests in children aged six to 59 months, stratified by sex and age group in central Côte d’Ivoire in 2012

	***Pf*** **positive by RDT**	
	**n/N (%)**	**95% CI**
**Sex**		
Male	112/164 (68.3)	61.1-75.5
Female	122/175 (69.7)	62.8-76.6
**Age (months)**		
6-11	22/46 (47.8)	32.8-62.8
12-23	52/80 (65.0)	54.3-75.7
24-35	54/82 (65.9)	55.4-76.3
36-47	54/69 (78.3)	68.3-88.2
48-59	52/62 (83.9)	74.5-93.3
Total	234/339 (69.0)	64.1-74.0

CI, confidence interval; *Pf*, *Plasmodium falciparum*; RDT, rapid diagnostic test.

### Prevalence and severity of anaemia

The prevalence and severity of anaemia, stratified by sex and age group, are summarized in Table 2. Anaemia, defined as Hb <11 g/dl, was recorded in 72.0% of the surveyed children. The prevalence of anaemia decreased with age (84.8% in children aged six to 11 months; and 64.5% in children aged 48-59 months). Slightly less than half of the children were moderately anaemic (Hb 7.0-9.9 g/dl; 47.5%). Severe anaemia (Hb <7 g/dl) was highest in the 24- to 35-month-old age group (4.9%), followed by the youngest age group (six-to 11-month-old; 4.6%). Boys and girls were equally affected by anaemia.

**Table 2:** Prevalence and severity of anaemia in children aged six to 59 months, stratified by sex and age group in central Côte d’Ivoire in December 2012

	**Mild anaemia**	**Moderate anaemia**	**Severe anaemia**	**Any anaemia**	
	**(Hb 10.0-10.9 g/dl)**	**(Hb 7.0-9.9 g/dl)**	**(Hb <7 g/dl)**	**(Hb <11 g/dl)**	
	**n/N (%)**	**n/N (%)**	**n/N (%)**	**n/N (%)**	**95% CI**
**Sex**					
Male	31/164 (18.9)	83/164 (50.6)	4/164 (2.4)	118/164 (72.0)	65.0-78.9
Female	43/175 (24.6)	78/175 (44.6)	5/175 (2.9)	126/175 (72.0)	65.2-78.7
**Age (months)**					
6-11	7/46 (15.2)	30/46 (65.2)	2/46 (4.6)	39/46 (84.8)	74.0-95.6
12-23	14/80 (17.5)	45/80 (56.3)	2/80 (2.5)	61/80 (76.3)	66.7-85.8
24-35	25/82 (30.5)	31/82 (37.8)	4/82 (4.9)	60/82 (73.2)	63.4-83.0
36-47	17/69 (24.6)	27/69 (39.1)	0/69 (0.0)	44/69 (63.8)	52.1-75.4
48-59	11/62 (17.7)	28/62 (45.2)	1/62 (1.6)	40/62 (64.5)	52.3-76.8
Total	74/339 (21.8)	161/339 (47.5)	9/339 (2.7)	244/339 (72.0)	67.2-76.7

CI, confidence interval; Hb, haemoglobin.

### Findings of the KAP survey

Key results of the KAP survey are shown in Table 3. While awareness of malaria was high (97.5%), knowledge of the disease was considerably lower. For example, when asked about causes of malaria, almost half of the mothers (47.6%) mentioned – without prompting – that mosquitoes transmit malaria. However, “being in the sun” was presented as the cause of malaria by more than half of the respondents (52.4%). Consequently, consistent knowledge on malaria transmission, i.e. malaria is solely transmitted through mosquito bites, was low (22.3%). Similarly, mothers’ knowledge pertaining to malaria prevention was poor. Possible prevention measures included “avoiding mosquito bites” (10.9%) and “avoid exposure to the sun” (30.6%). Most of the mothers reported to have taken intermittent preventive treatment (IPT) during their last pregnancy (87.2%).

**Table 3:** Knowledge, attitudes and practices related to malaria and health-seeking behaviour among mothers aged 15-49 years in central Côte d’Ivoire in December 2012

	**n/N (%)**	**95% CI**
**Malaria awareness**		
Ever heard about malaria	229/235 (97.5)	94.5-99.1
**Malaria transmission**		
Consistent knowledge on malaria transmission^a^	51/229 (22.3)	16.8-27.7
Knowledge that mosquitoes transmit malaria	109/229 (47.6)	41.1-54.1
Belief that exposure to sun can cause malaria	120/229 (52.4)	45.9-58.9
**Malaria symptoms**		
Fever stated as main malaria symptom	111/229 (48.5)	41.9-55.0
**Malaria prevention**		
Avoid mosquito bites	25/229 (10.9)	6.8-15.0
Avoid exposure to the sun	70/229 (30.6)	24.6-36.6
Mothers having received IPT_p_ during the last pregnancy	205/235 (87.2)	82.9-91.5
**Ownership and use of ITNs**		
**Mean number of ITNs per household (n)**	2.1	1.9-2.4
Households owning at least one ITN	182/235 (77.4)	71.6-82.6
Children aged <5 years who slept under an ITN the night preceding the survey^b^	235/382 (61.5)	56.4-66.4
**Indoor residual spraying**		
Households that had IRS within the 12 months preceding the survey	25/235 (10.6)	7.0-15.3
**Health seeking behaviour**		
Mothers who sought medical advice or treatment at a formal health facility the last time the youngest child had fever^c^	167/211 (79.1)	73.0-84.4
Mothers who ever consulted a traditional healer when child was sick^d^	90/231 (39.0)	32.6-45.6

^a^Knowing that malaria is only transmitted through mosquito bites; ^b^includes all children aged < five years; ^c^of all children who ever had fever; ^d^of all children who had ever been sick; CI, confidence interval; IPT_p_, intermittent preventive treatment during pregnancy; IRS, indoor residual spraying; ITN, insecticide-treated net.

Among the households visited, 77.4% owned at least one ITN and the mean number of ITNs per household was 2.1 (95% CI 1.9-2.4). Of the children below the age of five years, 61.5% slept under an ITN the night preceding the survey. The IRS coverage at household level was 10.6% (25 out of the 235 structures visited were sprayed within 12 months before the current survey).

Most of the mothers reported that they sought medical advice in a formal health facility during the last fever episode of their child (79.1%). Additionally, use of traditional medicine was quite common; among all respondents, 39.0% had consulted a traditional healer at some point with a sick child.

### Results from logistic regression analysis

Results from the logistic regression analysis for child and household attributed factors associated with *P. falciparum* infection and anaemia are summarized in Table 4. Age was significantly associated with *P. falciparum* infection, with younger age groups having a lower odds of infection (OR, 0.04; 95% CI 0.01-0.12 for the six to 11 months age group; OR, 0.14, 95% CI 0.05-0.38 for the 12-23 months age group; and OR, 0.17, 95% CI 0.06-0.47 for the 24-35 months age group) compared to the oldest age group (48-59 months). There was a strong association between *P. falciparum* infection and Hb level. Children who tested *P. falciparum* positive had a more than seven-fold higher odds of being anaemic (OR, 7.43; 95% CI 3.97-13.89) than their non-infected counterparts. Lack of access to a health care facility was positively associated with *P. falciparum* infection. Children living in villages with no health facility had a 5.59 times higher odds of *P. falciparum* infection. Consistent knowledge of respondents regarding transmission (i.e. malaria uniquely transmitted by mosquito bites) showed a negative association with *P. falciparum* infection among children (OR, 0.27; 95% CI 0.14-0.53).

**Table 4:** Results from multivariate logistic regression analysis of child, household and mother attributes associated with malaria and anaemia

	**Malaria**			**Anaemia**		
**Attribute**	**(%)**	**Adjusted OR**^**a**^**(95% CI)**	**p-value**	**(%)**	**Adjusted OR**^**a**^**(95% CI)**	**p-value**
**Sex**						
Male	68.3	1.00	-	72.0	1.00	-
Female	69.7	1.02 (0.58-1.80)	0.948	72.0	1.04 (0.61-1.78)	0.891
**Age (months)**						
6-11	47.8	0.04 (0.01-0.12)	<0.001*	84.8	7.98 (2.61-24.43)	<0.001*
12-23	65.0	0.14 (0.05-0.38)	<0.001*	76.3	3.10 (1.34-7.20)	0.008*
24-35	65.9	0.17 (0.06-0.47)	0.001*	73.2	2.51 (1.11-5.70)	0.028*
36-47	78.3	0.50 (0.17-1.47)	0.209	63.8	1.04 (0.48-2.30)	0.913
48-59	83.9	1.00	-	64.5	1.00	-
***P*.** ***falciparum*** **infection**						
RDT negative	-	-	-	49.5	1.00	-
RDT positive	-	-	-	82.1	7.43 (3.97-13.89)	<0.001*
**ITN use**^b^						
Yes	66.3	1.00	-	-	-	-
No	73.0	1.50 (0.83-2.69)	0.180	-	-	-
**Access to health care**						
Health facility	57.0	1.00	-	64.9	1.00	-
No health facility	80.5	5.59 (1.81-17.32)	0.003*	78.7	1.20 (0.68-2.11)	0.535
**ITN distribution**^c^						
Not received	63.5	1.00	-	-	-	-
Received	77.9	0.47 (0.15-1.50)	0.203	-	-	-
**Education of mother**						
Some formal education	64.0	1.00	-	72.1	1.00	-
No formal education	73.6	1.15 (0.64-2.08)	0.635	71.9	0.83 (0.48-1.43)	0.503
**Consistent knowledge of mother**^d^						
No	76.6	1.00	-	-	-	-
Yes	45.8	0.27 (0.14-0.53)	<0.001*	-	-	-

^a^Multivariate logistic regression odds ratio (OR); ^b^children aged six to 59 months who have slept under an ITN the night preceding the survey according to the mother; ^c^households that received an ITN during the private sector ITN distribution in June 2012; ^d^knowing that malaria is only transmitted through mosquito bites; *significant p-value; CI, confidence interval; Hb, haemoglobin; ITN, insecticide-treated net; RDT, rapid diagnostic test.

Children in sentinel sites who received an ITN through the private sector distribution campaign six months prior to the current cross-sectional survey (i.e. Bonikro, Bandamakro, Petit Bouaké and Konankro) were at about half the odds of *P. falciparum* infection when compared to the other sentinel sites (OR, 0.47; 95% CI 0.15-1.50). Sleeping under an ITN the night preceding the survey and educational status of the mother were found to be non-significant (p >0.05) for all attributes.

Child age was found to be strongly associated with anaemia. Children younger than 35 months were at a significantly higher odds of anaemia than their older counterparts. The odds for anaemia in children aged six to 11 months was eight times higher than for the oldest age group (OR, 7.98; 95% CI 2.61-24.43).

## Discussion

This study found high prevalences of *P. falciparum* (69.0%) and anaemia (72.0%) in children aged six to 59 months and limited knowledge on malaria transmission and prevention among children’s mothers in communities located in the zone of influence of the BGM project area in central Côte d’Ivoire. The comparison of the observed *P. falciparum* prevalence with reports in previous surveys from central and south-central Côte d’Ivoire [16-18,24,25] underscores that malaria is highly endemic but that small-scale heterogeneity is considerable. For example, Koudou *et al.*[16] conducted several cross-sectional entomological and parasitological surveys in two villages in central Côte d’Ivoire (distance between the villages is approximately 80 km) to better understand the transmission of malaria. In surveys conducted in August 2005, the authors found prevalences of 62% and 82% in children aged six years and below in the two study villages, as determined by Giemsa-stained blood films examined microscopically. Righetti *et al.*[17,26] reported *P. falciparum* prevalences of 45.3% and 78.2% (using RDTs) among two cohorts of children consisting of (i) six- to 23-month-old infants and (ii) six- to eight-year-old schoolchildren, respectively, in three settings of the Taabo health and demographic surveillance system in south-central Côte d’Ivoire in a cross-sectional survey done in April 2010. Comparing the RDT results presented here for the same age group with data from the central-western region of Côte d’Ivoire obtained during the 2011/2012 national DHS [18] revealed a considerable difference: 69% (current study) *versus* 43% (DHS). The observed differences might partly be explained by seasonality [27,28]. However, the local social-ecological contexts, potentially affected by population influx, might also play a role. For example, several studies reported an adaptation of *An. gambiae* to polluted water bodies [8,29].

Interestingly, *P. falciparum* prevalence was found to be associated with three non-behavioural factors (i.e. anaemia status, host age and access to health care) and one behavioural factor (i.e. consistent knowledge of respondents regarding malaria transmission). The association between anaemia and malaria is widely acknowledged in the literature [22,30]. In the current study area, the sentinel sites that do have health facilities are somewhat more urbanized. Hence, access to health care is likely to be linked to other socio-economic and health systems factors that differ between the urban and rural settings. The fact that knowledge of malaria transmission by mothers is associated with lower odds of *P. falciparum* infection may imply that mothers who are aware of the risk take measures to protect their children, or have better treatment-seeking practices.

Anaemia was highly prevalent in the six- to 59-month-old children in this survey (72.0%). A similarly high prevalence was found in the same age group during the recent DHS at the national level (75%), but it is somewhat lower than the regional average for the central-western region (83%) [18]. Righetti *et al.*[17] reported an anaemia prevalence of 78% among six- to 23-month-old infants and 47% among schoolchildren aged six to eight years. Anaemia is multifactorial, with malnutrition, intestinal parasite infections (most importantly hookworm) and malaria being the key drivers [22,31,32]. The current study did not investigate concurrent helminth infections and micronutrient deficiencies, and hence it is not possible to quantify the differential contribution of these factors to the high rate of anaemia. The observed association of anaemia with child age has been reported before, and the proposed, underlying mechanism is that iron requirements are related to growth rate, and hence iron demand declines with age [25,33].

In Côte d’Ivoire, ITN coverage, defined as households owning at least one ITN, has been intensely scaled up from less than 10% in 2005 to 60% in 2011 [14]. After the most recent distribution campaign in 2011/2012, preceding the current survey in December 2012, the coverage of the central-western region had further increased to 77.4% according to DHS data [18]. The study area had a comparable household coverage of ITNs with 76.7%, though the utilization among children below the age of five years lagged behind in both the current survey (61.5%) and the DHS (50.6% in central-western region) [18,34]. It is interesting to note that no clear association between ITN ownership and/or use and *P. falciparum* infection prevalence was found, but this may be explained by other confounding factors. The KAP survey highlighted important inadequacies in malaria-related knowledge, its transmission and prevention. Misconceptions on causes of malaria are common, as revealed here with more than 50% of the interviewed mothers mentioning working in the sun. On the other hand, the correct cause (i.e. transmission of malaria is uniquely due to mosquito bites) was stated by less than half of the mothers. Taken together, these findings clearly indicate that intervention campaigns of any kind (e.g. ITN distribution) and introduction of RDTs should go hand-in-hand with setting-specific information, education and communication (IEC) strategies in order to enhance the impact of ITNs.

In July 2012, five months prior to this survey, an entomology and insecticide resistance survey found that *An. gambiae s.s.* is the main vector in the study area. Wild caught female mosquitoes (n = 211) from Oumé and Hiré towns were exposed for one hour to either 0.05% deltamethrin, 4% malathion, 0.1% bendiocarb or 4% dichlorodiphenyltrichloroethane (DDT) [35]. Mortality 24 hours after exposure was 15.1% for deltamethrin, 10.4% for malathion, 6.9% for bendiocarb and 10.5% for DDT. Further, knock-down resistance, both east and west resistance alleles, were found in 83% and 96% of mosquitoes (n = 111), respectively, and G119S mutations in 37%. These findings confirmed earlier indications of high resistance to insecticides in Côte d’Ivoire and in neighbouring countries [36-38]. Based on these results, IRS was initiated by BGM, which led to a relatively high IRS coverage in the study area (10.6%) compared to the regional average for the central-western region (0.5%). All structures surveyed in Bonikro and Bandamakro were sprayed through the BGM IRS programme. Outside these sites, only two of the interviewed households reported that they had undergone IRS, resulting in an overall coverage of 0.9%, which is comparable with data from the recent DHS. The combined protective effects of IRS and ITNs have been documented in the literature with some inconsistency [39,40]. Corbel and colleagues [41], for example, concluded that IRS combined with ITNs in an endemic, high pyrethroid-resistance area in Benin, had no beneficial effect. However, the combination of IRS and case management has proven successful in a malaria control programme on Bioko Island, Equatorial Guinea [42]. A significant reduction in malaria prevalence (42% less infections within two years) has been observed in locations where spray intervals and coverage were optimized. The programme demonstrated the benefits of collaboration between the private sector (Marathon Oil) and local government (the Government of Equatorial Guinea).

## Conclusion

The surveyed communities in a major mining area of central Côte d’Ivoire are significantly affected by malaria and anaemia, whilst access to health care, IEC strategies and prevention measures are limited. The effectiveness and sustainability of malaria control interventions can be enhanced through intersectoral collaboration, including public-private partnerships. The private sector has direct business interests and social cooperate responsibility to support malaria control in the project area. The data presented here provide a rationale for setting-specific interventions. Follow-up surveys pertaining to *Plasmodium* infection and anaemia in the PACs will allow for long-term monitoring of potential project-related impacts pertaining to environmental and socio-economic changes and the effect of malaria control interventions, such as ITN distribution, IRS or IEC activities [20,43].

## Competing interests

This study received financial support from Newcrest Mining Limited. The funder had no role in the study design, data collection and analysis, decision to publish or preparation of the manuscript. RMY and PAY were employees of Newcrest Mining Limited at the time of the survey. All other authors declare that they have no competing interests.

## Authors’ contributions

AMK conceived the study design and data collection of the malaria and anaemia prevalence and knowledge, attitudes and practices surveys. AMK and CA managed the field activities. MJD was the overall study coordinator. AMK and MO conducted the statistical analysis. AMK and MSW drafted the manuscript. RMY was the medical director of the study, assisted in obtaining ethical clearance and was in charge of sensitizing the local health authorities. RMY and PAY supported the sensitization of the communities and assisted with data collection. CA, MJD, MO and JU contributed to the interpretation of the data, manuscript writing and revisions. All authors read and approved the final manuscript.
